# Application of *Scutellariae radix*, *Gardeniae fructus*, and Probiotics to Prevent *Salmonella enterica* Serovar Choleraesuis Infection in Swine

**DOI:** 10.1155/2013/568528

**Published:** 2013-02-26

**Authors:** Chiung-Hung Chang, Yueh-Sheng Chen, Ming-Tang Chiou, Chiu-Hsian Su, Daniel S. Chen, Chin-En Tsai, Bi Yu, Yuan-Man Hsu

**Affiliations:** ^1^School of Chinese Medicine, China Medical University, Taichung 404, Taiwan; ^2^Department of Traditional Chinese Medicine, Taichung Veterans General Hospital, Taichung 407, Taiwan; ^3^Department of Veterinary Medicine, National Pingtung University of Science and Technology, Neipu, Pingtung 912, Taiwan; ^4^Department of Biological Science and Technology, College of Life Sciences, China Medical University, 91, Hsueh-Shih Road, Taichung 404, Taiwan; ^5^Department of Biochemistry, College of Agriculture and Life Sciences, University of Wisconsin, Madison, WI 53715, USA; ^6^Department of Animal Science, College of Agriculture and Natural Resources, National Chung Hsing University, Taichung 402, Taiwan

## Abstract

*Salmonella enterica* serovar Choleraesuis, a host-adapted pathogen of swine, usually causes septicemia. Lactic acid bacteria (LAB) strains have been widely studied in recent years for their probiotic properties. In this study, a mouse infection model first screened for potential agents against infection, then a pig infection model evaluated effects of LAB strains and herbal plants against infection. *Scutellariae radix* (SR) and *Gardeniae fructus* (GF) showed abilities to reduce bacteria shedding and suppressing serum level of TNF-**α** induced by infection in swine. Bioactivities of SR and GF were enhanced by combining with LAB strains, which alone could speed up the bacteria elimination time in feces and boost immunity of infected pigs. Baicalein and genipin exhibited stronger cytotoxicity than baicalin and geniposide did, as well as prevent *Salmonella* from invading macrophages. Our study suggests LAB strains as exhibiting multiple functions: preventing infection, enhancing immunity to prepare host defenses against further infection, and adjusting intestinal microbes' enzymatic activity in order to convert herbal compounds to active compounds. The SR/GF-LAB strain mixture holds potential infection-prevention agents supplied as feed additives.

## 1. Introduction

*Salmonella* infection of humans and animals is still a severe health problem worldwide [1], *S*.* enterica* a key gastrointestinal pathogen responsible for food-borne diseases. In particular, *S. enterica* serovar Choleraesuis, a host-adapted pathogen of swine, sometimes causes necrotizing enterocolitis as well as septicemia characterized by hepatitis, pneumonia, and cerebral vasculitis [2]. *S. Choleraesuis* also produces systemic infection in humans. It is a major serovar for salmonellosis transmitted from animals to humans [3]. Yet antibiotic treatments have served to eliminate *Salmonella *infection. As with other bacterial pathogens, antibiotic resistance to *Salmonella *is a growing problem for eradication. Finding safe and efficient prevention to alleviate the need or even replace antibiotics for cleaning infection looms is a vital task.

Lactic acid bacteria (LAB) strains have been widely studied in recent years for their probiotic properties and are used as feed supplements [4, 5]. A number of studies show LAB strains bolstering immunity and warding off infection by gastrointestinal pathogens [4–7]. They can also act as immunomodulators or adjust composition and activity of intestinal microbes [8]. Intestinal microbial community can also play a role as probiotics [9]. Prior studies indicated that intestinal microflora could transform herbal components into bioactive compounds, for example, convert ginsenoside Rb1 to compound K, which is more active and subsequently absorbed [10–13]. We first screened seven herbs via mice infection model for potential agents, that is, *Scutellariae radix* (SR), *Gardeniae fructus* (GF), *Houttuyniae herba*, *Taraxaci herba*, *Glycyrrhizae radix*, *Puerariae radix*, and *Rhizoma dioscoreae*. LAB strains and potential herbal materials as pig feed supplements allowed the study of their preventive effects against *Salmonella* infection, thus offering an alternative way to improve immunity and digestive abilities of animals by using combinations of LAB strains and herbs as feed additives.

## 2. Materials and Methods

### 2.1. Preparation of Herbal Extracts

All herbal materials were purchased from Ko Da Pharmaceutical Co., Ltd. at Taoyuan, Taiwan: *Scutellariae radix* (SR), *Gardeniae fructus* (GF),* Taraxaci herba*, *Houttuyniae herba*, *Glycyrrhizae radix*, *Puerariae radix*, and *Rhizoma dioscoreae*. Plant materials were finely powdered and extracted using distilled water at 100°C for 1 hr (plant: water = 1 : 10, w/v). With insoluble matter removed by filtration, filtrate was concentrated in vacuum and lyophilized to yield residue. Percentages of indicator compounds in each original herbal plant were confirmed by high-performance liquid chromatogram (HPLC) by Ko Da Pharmaceutical Co., Ltd. and listed in Table 1. Water extracts were used for mice infection model; plant materials of SR and GF pulverized to fine powder and passed through an 80-mesh sieve. Finely powered plant materials were used for pig infection model. For *in vitro* tests, baicalin and baicalein were purchased from Sigma-Aldrich Co. LLC (St. Louis, MO, USA); geniposide and genipin were from Wako Pure Chemical Industries, Ltd. (Osaka, Japan) and Challenge Bioproducts Co., Ltd. (Taichung, Taiwan), respectively.

### 2.2. Bacterial Strains and Culture Conditions

*S. Choleraesuis* (ATCC 13312) reference strain was obtained from the American Type Culture Collection (ATCC). Strain SC37 was collected from a carrier pig to be used as inocula for infecting animals; is serotype identification applied as follows. Antiserum to detect O and H antigens was purchased from S&A Reagents Lab Ltd. (Bangkok, Thailand) and from Denka Seiken Co., Ltd. (Tokyo, Japan), respectively. Isolate was analyzed according to Kaufmann-White scheme and serotyping protocols developed by Centers for Disease Control and Prevention (Atlanta, GA, USA) [14]. Bacterial inocula were grown in Luria Bertani (LB) broth to log stationary phase at 250 rpm and at 37°C for 8 hr to an OD_600 _nm of 0.8. After centrifugation at 8,000 ×g, bacterial pellet was resuspended in phosphate-buffered saline (PBS) and adjusted to final concentration of 10^10^ colony forming units (CFUs)/mL in PBS. Two lactic acid bacteria (LAB) strains, *Lactobacillus acidophilus* strain LAP5 [4] and *L. reuteri* Pg4 [15], were isolated from the gastrointestinal tract of swine and broilers, respectively, as described previously. LAB strains were grown in deMan-Rogosa-Sharpe (MRS) broth (Merck, Darmstadt, Germany) for 24 hr at 37°C. Whole cells were obtained by centrifugation at 6000 ×g for 10 min and washed twice with sterilized PBS (pH 7.2). Mix-LAB strains LAP5 and Pg4 were combined in a ratio of 1 : 1 used for swine experiments. LAB culture preparation was then lyophilized and stored at 4°C until later required. The bacterial count of the LAB strain powder was approximately 10^9^ CFU/g. 

### 2.3. Antimicrobial Activity

Minimum inhibitory concentration (MIC) of extract or compound was tested by twofold serial dilution. Test extracts were first dissolved in PBS, then incorporated into Muller-Hinton broth to obtain concentration of 100 mg/mL and serially diluted to attain 50, 25, 12.5, 6.25, or 3.125 mg/mL. Test compounds were dissolved in dimethyl sulfoxide (DMSO) to obtain concentration of 20 mM and serially diluted to achieve 10, 5, 2.5, 1.25, and 0.625 mM. A volume of 100 *μ*L of bacterial suspension adjusted previously at concentration 10^6^ CFU/mL was added to 100 *μ*L of the herbal extracts or compounds, then an additional tube containing broth only used as a negative control was added. All test tubes and control were incubated at 37°C for 18–24 hr, after which the tube containing the lowest concentration of extract showing no visible growth was considered as MIC. Further, concentrations showing total inhibition of visual bacteria growth were identified. 50 *μ*L of each culture broth transferred onto agar plates and incubated for 18–24 hr at 37°C. Complete absence of growth of bacterial colonies on the agar surface in the lowest concentration of sample was defined as minimum bactericidal concentration (MBC). Each assay in this experiment was replicated three times.

### 2.4. Mice Infection Model

Male BALB/c mice weighed about 20–22 g, obtained from the National Laboratory for Animal Breeding and Research Center, were maintained in specific pathogen-free (SPF) condition and used at 8–10 weeks of age. They were housed in an air-conditioned room at 25 ± 2°C with relative humidity 40–70% and 12-hour light/dark cycle and were fed with tap water and standard laboratory rodent diet. All animal experimental protocols were approved by the Institutional Animal Care and Use Committee of China Medical University (approval no.: 99-156-B); experiments were performed according to the institutional ethical rules and laws. Mice were fed basal diet for a week before being used in the study. On treatment day 0, mice were randomly allocated into nine groups of ten mice. Herbal extract was dissolved in water and given orally at a dose of 5 mg/mice for seven days via stomach tube. Control and water groups were given the basal diet and were fed by water instead. After the last dose, herbal treated and watered mice were challenged orally with 10^10^ CFU of strain SC37 per mouse. Clinical signs were monitored, body weight measured, and feces of each individual were collected daily for four days after challenge. Sera, intestines, livers, and spleens were collected from all groups on day 4 after challenge; all mice were humanely euthanized.

### 2.5. Pig Infection Model

In all, 35 crossbreds weaned pigs weighing about 8–10 kg were obtained from the Animal Technology Institute Taiwan, maintained in SPF condition, and used at 28 days of age. All animal experimental protocols were approved by the Institutional Animal Care and Use Committee of National Pingtung University of Science and Technology (approval no.: NPUST-IACUC-100-004); experiments were performed according to the institutional ethical rules and laws. SPF pigs were housed in an environmentally controlled isolation facility with filtered air and supplied with sterilized food and water. They were fed basal diet for three days before study. On treatment day 0, pigs were randomly allocated into seven of five groups (control, water, Mix-LAB, SR, SR + Mix-LAB, GF, and GF + Mix-LAB groups). Herbs were mixed in a diet with concentration 0.1%, and was Mix-LAB added to final amount of 10^6^ CFU per gram. The dosage of herbs and Mix-LAB supplied in diet was based on previous studies [16, 17]. Both control and water groups were given basal diet and were fed by water instead. After 10 days of experimental diet, treated and water groups were challenged orally with 10^10^ CFU strain SC37 per pig. Clinical signs were monitored for six days after challenge. During this time, pigs were fed with experimental diets. Body weight, body temperature, and feces of each pig were measured daily for six days. Sera and intestines were collected from all pigs on the sixth day after challenge; pigs were then humanely euthanized by electrocution.

### 2.6. Shedding of Challenge Bacteria from Feces

Fecal samples were collected at 1–6 days in pig trials after administering strain SC37, and numbers of *Salmonella* per gram in feces were determined. Aliquots (100 *μ*L) of fecal suspensions were serially diluted in PBS, plated on duplicate* Salmonella*-*Shigella* (SS) agar plates (Difco, NJ, USA), and incubated overnight at 37°C. Typical colonies were counted for plates containing between 30 and 300 colonies. *S. Choleraesuis* was confirmed by PCR assay, as described in a previous report [18].

### 2.7. Measurement of Bacteria Load in Blood and Organs

After the animals were sacrificed, their livers, spleens, intestines were aseptically removed, and blood was collected to determine bacteria load. Organs were homogenized in 1.0 mL of sterile saline with the aid of a tissue homogenizer maintained at 4°C, aliquots of homogenate processed for CFU counts.

### 2.8. Enzyme-Linked Immunosorbent Assay (ELISA) for Evaluation of Cytokine Levels

Tumor necrosis factor-*α* (TNF-*α*), interferon-*γ* (IFN-*γ*), and interleukin-8 (IL-8) in peripheral blood were detected with an ELISA kit (BioLegend, Inc., San Diego, CA, USA) according to instructions of manuals.

### 2.9. Cell Viability Assay

The murine monocyte/macrophage cell line RAW 264.7 (ATCC TIB-71) was obtained from ATCC; RAW264.7 cells were seeded onto 24-well plates at a density of 1 × 10^5^ cells/well for 24 hr. Indicated concentrations of baicalin, baicalein, geniposide, or genipin were then added to cells, while only adding 0.1% DMSO in the control group, and grown at 37°C for 24 hr. To ascertain cell viability, trypan blue exclusion protocol was used. Briefly, about 10 *μ*L of cell suspension in PBS (pH 7.4) was mixed with 10 *μ*L of trypan blue; stained (dead cells) and unstained (live cells) were counted by a hemocytometer. Viability represented the percentage of cell survival after treatment.

### 2.10. Invasion Assay

RAW264.7 cells were treated with baicalin, baicalein, geniposide, or genipin for 0.5 hr before infection in antibiotic-free Dulbecco's Modified Eagle Medium (DMEM) supplemented by 10% fetal bovine serum (FBS), then cocultured in PBS-resuspended SC37 at multiples of infection (MOI) 10. Designated concentration of each indicator compound was then added to cells, while only adding 0.1% DMSO in the infection group. Cell-associated bacteria were quantified 0.5 hr after infection, then treated by 100 *μ*g/mL of gentamicin for 1.5 hr. Cell culture supernatants were removed gently; cells were washed with PBS, and osmotic lysis was performed to quantify total bacteria. For this purpose, sterile water was added to infected cells after washing, and cell lysates were resuspended with PBS, and bacterial numbers were derived by plating serial dilution on SS agar plates. Invasion activity was calculated as triplicate mean, and results were expressed as percent relative invasion of RAW264.7 cells were compared with infection group.

### 2.11. Statistical Analysis

Differences between treated and control groups in mean values were rated by Student's *t*-test, using SPSS software (SPSS, Inc., Chicago, IL, USA).

## 3. Results

### 3.1. Antibacterial Activity of Seven Herbal Extracts

In order to screen for the herbs with bactericidal property against *S. Choleraesuis* infection, seven herbal plants were tested.  Table 2 summarizes their MBCs against *S. Choleraesuis*, ATCC 13312, and SC37; only SR displayed antibacterial activity against *Salmonella* strains tested. MBCs against reference strain and SC37 were 6.25 and 25 mg/mL, respectively, but bactericidal activities of other herbal extracts exceeded 50 mg/mL. SR showed moderate antibacterial activities against *S. Choleraesuis*, and the reference strain was more susceptible to SR than to clinical isolate.

### 3.2. Effects of Herbal Extracts on Preventing *S. Choleraesuis* Infection in Mice

**Figure 1:**
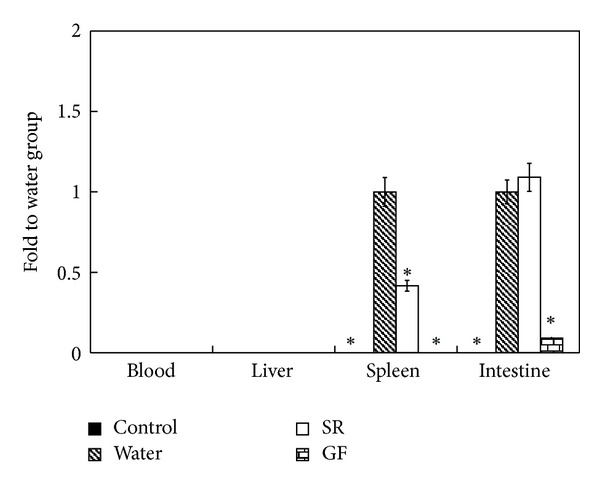
Effects of SR and GF supplements on *Salmonella* load in blood, livers, spleens, and intestines on the fourth day after challenge. Bacterial load in organs of water group was set as 100%; results were expressed as mean ± standard deviation (10 mice per group). Asterisks indicate significant difference from water group (*P < 0.05).

**Figure 2:**
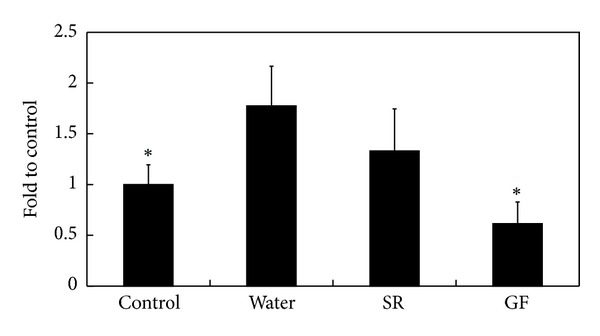
Effects of SR and GF supplements on SC37-induced IFN-*γ* expression in serum on the fourth day after challenge. IFN-*γ* level of control group set as 100%. Results are expressed as mean ± standard deviation (10 mice per group). Asterisks indicate significant difference from water group (*P < 0.05).

Supplemental dosage of herbal medicine used in this study was 5 mg/mouse, that is, 0.1% of daily regimen for each mouse. Clinical signs were observed, and body weight was measured. SC37 infection did not cause significant symptoms and did not affect body weight in BALB/c mice (data not shown). Yet no herbal plant treatment could reduce level of *Salmonella* shedding in feces caused by challenge (data not shown); only SR and GF could eliminate *S. Choleraesuis* in organs of infected mice and significantly decrease *Salmonella* load in spleens. GF treatment reduced amount of *Salmonella* in both spleen and intestine (Figure 1). No bacteria were harvested from blood and livers of infected mice. IFN-*γ* levels were also monitored. GF could significantly decrease *S. Choleraesuis*-induced IFN-*γ* expression in serum (Figure 2), but not the other six herbs. Hence, GF could suppress inflammation induced by *Salmonella* infection. Given the above, SR and GF might offer an alternative way to prevent infection.

### 3.3. Effects of SR, GF, and Mix-LAB on Preventing *S. Choleraesuis* Infection in Pigs

Based on data in mice model, SR and GF had the best bioactivities in eliminating bacteria and suppressing inflammation induced by infection. Bioactivities of SR and GF were further evaluated by pig infection model; probiotics as feed supplements are commonly used in livestock diets. This study applied two LAB strains (Mix-LAB) for investigating their probiotic property along with herbs.

**Figure 3:**
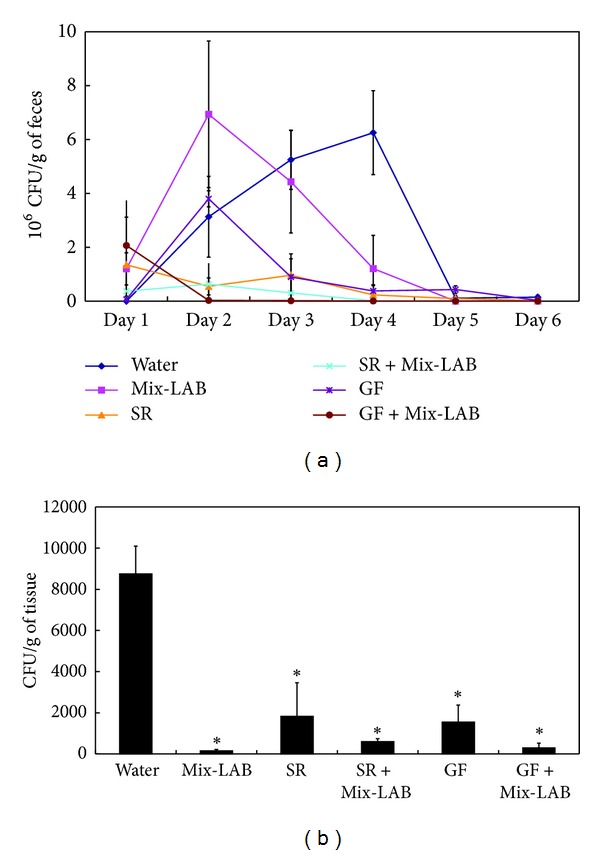
Effects of SR, SR + Mix-LAB, GF, and GF + Mix-LAB supplements on bacteria load in (a) feces during six-day period after challenge and (b) intestines on the sixth day after challenge. Results are expressed as mean ± standard deviation (five pigs per group). Asterisks indicate significant difference from water group (*P < 0.05).

**Figure 4:**
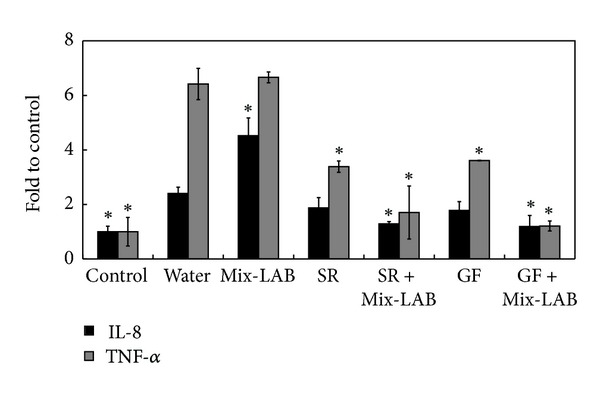
Effects of Mix-LAB, SR, SR + Mix-LAB, GF, and GF + Mix-LAB supplements on SC37-induced IL-8 and TNF-*α* expressions in serum on day 6 after challenge. IL-8 and TNF-*α* levels of control group set as 100%. Results are expressed as mean ± standard deviation (five pigs per group). Asterisks indicate significant difference from water group (*P < 0.05).

After a 10-day supplement, pigs were challenged with the clinical isolate SC37. This study was designed to show the interaction and bioactivity of herbs and probiotics as feed supplements in preventing *Salmonella* infection. We were not intent on producing server sickness in pigs. The infection did not affect the body weight or cause fever, but only induced diarrhea and mild inflammatory responses in pigs. Mix-LAB, SR, and GF supplements could all reduce the levels of diarrhea caused by infection. Furthermore, SR, SR + Mix-LAB, GF, and GF + Mix-LAB groups could also dramatically reduce the *Salmonella* shedding in feces (Figure 3(a)) and intestines (Figure 3(b)). The amount of *Salmonella* fecal shedding in the group fed with SR + Mix-LAB and GF + Mix-LAB was even lower by 68% and 80% than that in the groups fed with SR and GF alone. SR, SR + Mix-LAB, GF, and GF + Mix-LAB could all decrease SC37-induced IL-8 levels in serum. And SR + Mix-LAB and GF + Mix-LAB could reduce both IL-8 and TNF-*α* expressions in serum, closer to the levels of control groups. Duration of *Salmonella* fecal shedding in pigs fed with Mix-LAB was shorter than that in the infection group. Still, as of day 6 after challenge, bacteria load in intestines of the Mix-LAB group tallied the lowest and was reduced by 97% compared with the infection group. Mix-LAB supplement elevated immune abilities in swine. Serum levels of TNF-*α* and IL-8 were still high in Mix-LAB group, even with most *Salmonella* shed by that time (Figure 4).

### 3.4. Bioactivities of Baicalin, Baicalein, Geniposide, and Genipin *In Vitro*

Based on the data above, SR and GF were bioactive, eliminating bacteria in intestines and suppressing inflammation induced by infection. Since both have shown aglycones as metabolites wrought by intestinal microbes [19, 20], we evaluated bioactivities of baicalin, baicalein, geniposide, and genipin. MBCs of baicalin and baicalein against *S. Choleraesuis* strains were both 5 mM, whereas MBCs of genipin against test strains were both 10 mM; those of geniposide against *S. Choleraesuis* strains were above 10 mM (Table 2). Baicalin and baicalein showed stronger anti-*S*. *Choleraesuis* activity than geniposide and genipin. Furthermore, RAW264.7 viability was inhibited by baicalin with IC_50_ of 475 *μ*M and was unaffected by geniposide at concentrations less than 3 mM. By contrast, IC_50_ of baicalein and genipin dropped as low as 155 *μ*M and 1.42 *μ*M, respectively. Their aglycones exhibited stronger cytotoxicity.

**Figure 5:**
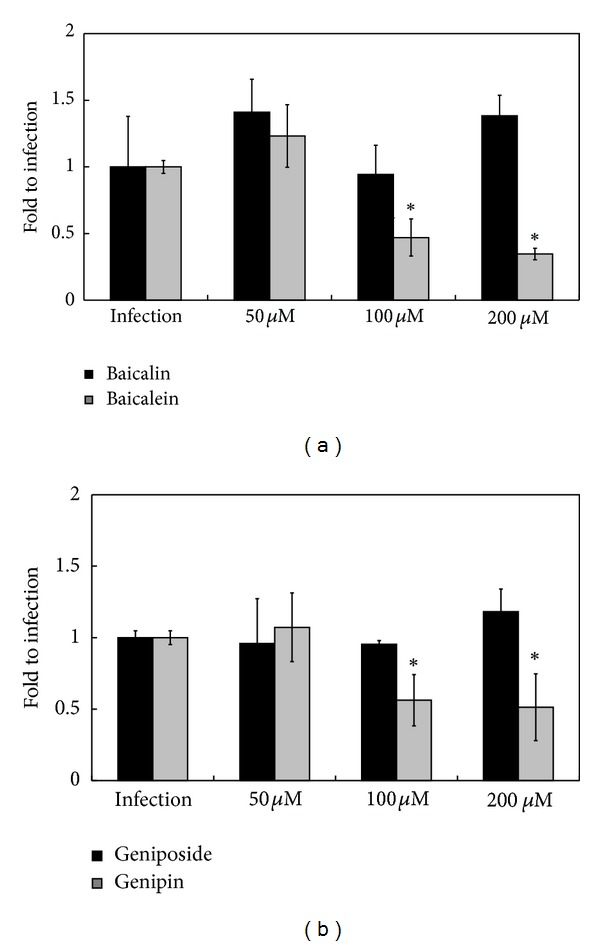
Effects of (a) baicalin and baicalein and (b) geniposide and genipin on SC37 invasion in RAW264.7 cells. Results are expressed as mean ± standard deviation from three independent trials. Asterisks indicate significant difference from infection group (*P < 0.05).

Abilities of these compounds to prevent SC37 from invading macrophages were scrutinized. Neither baicalin nor geniposide could inhibit *Salmonella* invasion of macrophages, even at concentration of 200 *μ*M. However, 100 *μ*M of baicalein and genipin could prevent 52% and 44% of bacteria invading cells, respectively (Figure 5), this decrease in a dose-dependent manner. Data also suggest aglycones with stronger ability to impede *S. Choleraesuis* invasion.

## 4. Discussion

*Lactobacillus* spp., *Bifidobacterium* spp., *Enterococcus* spp., and many other intestinal microbes are proposed as probiotics. LAB strains show beneficial effects in health promotion for a host [21]. Some LAB strains as feed supplements can prevent gastrointestinal infection [22–24]. Mechanisms considered for such probiotic function include enhancing immune response of the host [21] and protecting colonization by pathogens for attachment [5]. Several studies have indicated that probiotics could play immunomodulatory roles in reducing *Salmonella* colonization in mice and chickens [4, 7, 23, 24]. In addition, intestinal microflora offers ability to convert and then alter bioactivity of herbal compounds [25, 26].

This study aimed for a combination of LAB and herbal plants as feed additives to prevent *S. Choleraesuis* infection in swine. The mouse infection model screened for infection preventive agents; pig infection model evaluated effects of target herbs and LAB strains against infection. Seven herbal plants served to evaluate their efficacy in preventing *Salmonella* infection. They grow locally in Taiwan and hold antibacterial potential, seeing wide use in folk medicine. SR alone showed antibacterial activity *in vitro* against *S. Choleraesuis*. Mice are not common models to establish such infection. However, it is a relatively accurate way to evaluate bioactivity of herbs *in vivo* in order to screen for preventive agents. We managed to monitor several useful indicators affected by challenge, for example, *Salmonella* shedding in feces, *Salmonella* load in organs, and levels of serum IFN-*γ*. In mouse infection model, both SR and GF could eliminate *S. Choleraesuis* in spleens. GF could also decrease the bacteria load in intestines and infection-induced IFN-*γ* expression in serum. However, SR did not suppress the load of *S. Choleraesuis* in intestines; therefore, the serum levels of IFN-*γ* were not suppressed in serum significantly. Biotransformation in mice's intestines might not be active enough to process SR into active compounds, therefore, affecting the elimination of bacteria from intestines.

SR sees wide use in traditional Chinese medicine [19, 27, 28], for example, antiallergy [29–31], antioxidation [32, 33], -inflammation [28, 33], and anticancer [34, 35] treatments. It has shown strong antimicrobial effect *in vitro* [27, 36, 37]. GF functions as herbal remedy to treat liver and gall bladder disorders such as hepatitis and acute jaundice, as well as inflammation and fever in Chinese medicine for many years [38]. GF has the effective biological actions, including protective activity against oxidative damage [39], as well as cytotoxic [40], anti-inflammatory [41, 42], and fibrinolytic activities [43]. However, no study refers to its anti-*Salmonella* activity.

SR and GF were proved to be able to reduce bacteria shedding and decrease inflammation induced by challenge in swine in a significant manner. Likewise, bioactivities of SR and GF were enhanced combining with Mix-LAB. In our pig infection experiments, Mix-LAB did boost immunity of infected pigs, even with *S. Choleraesuis* already eliminated from the body. Results concurred with previous findings [4, 7] and indicated LAB strains adjusting immunity to prepare host defense against further infection. Mix-LAB could also speed up bacteria elimination time. Maximum bacterial shedding in the group treated with Mix-LAB was two days earlier than in the infection group, suggesting LAB strains adhere to gastrointestinal epithelium and providing a barrier against pathogen colonization [21, 22, 24].

For further identification of active compounds in herbs, bioactivities of indicator compounds baicalin and geniposide and their aglycones baicalein and genipin were studied *in vitro*. The aglycones revealed significant effects in bactericidal activity and cytotoxicity. Prior study demonstrated similar phenomena. It was reported that antimetastatic or anticarcinogenic activity of ginsenoside Rb1 could not show until biotransformation by intestinal bacteria [10–12]. Baicalin, crocin, amygdalin, geniposide, puerarin, ginsenoside Re, glycyrrhizin, hesperidin, and poncirin must be biotransformed by human fecal microflora in order to manifest cytotoxicity against tumor cells [26]. We attained conversion by supplying herbs and LAB mixture in diet and by using an infection model to study the effects. LAB strains could transform compounds in SR and GF, meaning that the amount of *Salmonella* fecal shedding and inflammation in the group fed with probiotics and herbs was even lower than those fed with herbs alone. In our study, LAB strains thus showed multiple functions for preventing infection, enhancing immunity to prepare host defense for infection, and adjusting enzymatic activity of intestinal microbes in order to convert bioactivity of compounds. Since SR and GF herbs grow in Taiwan, this study would also improve economic value of both plants as infection preventives for farm animals.

## Figures and Tables

**Table 1:** Concentrations of indicator compounds in herbal extracts used in this study.

Herbal extract	Indicator compound	mg/g
*Scutellariae radix *	Baicalin	89.74
*Gardeniae fructus *	Geniposide	40.15
*Houttuyniae herba *	Quercetin	0.07
*Taraxaci herba *	Chlorogenic acid	0.18
*Glycyrrhizae radix *	Glycyrrhizic acid	27.91
*Puerariae radix *	Puerarin	29.40
Rhizoma dioscoreae*	None	—

*No indicator compound available.

**Table 2:** MBCs of herbal extracts and compounds against *S. Choleraesuis* strains.

Herbal extract	ATCC 13312	SC37 isolate
*Scutellariae radix *	6.25 mg/mL	25 mg/mL
*Gardeniae fructus *	>50 mg/mL	>50 mg/mL
*Houttuyniae herba *	>50 mg/mL	>50 mg/mL
*Taraxaci herba *	>50 mg/mL	>50 mg/mL
*Glycyrrhizae radix *	>50 mg/mL	>50 mg/mL
*Puerariae radix *	>50 mg/mL	>50 mg/mL
*Rhizoma dioscoreae *	>50 mg/mL	>50 mg/mL
Baicalin	5 mM	5 mM
Baicalein	5 mM	5 mM
Geniposide	>10 mM	>10 mM
Genipin	10 mM	10 mM
